# Freshwater insects of different feeding guilds ingest microplastics in two Gulf of Guinea tributaries in Nigeria

**DOI:** 10.1007/s11356-020-08763-8

**Published:** 2020-06-23

**Authors:** Emmanuel O. Akindele, Sonja M. Ehlers, Jochen H. E. Koop

**Affiliations:** 1 Department of Animal Ecology, Federal Institute of Hydrology, Am Mainzer Tor 1, 56068 Koblenz, Germany; 2 Department of Zoology, Obafemi Awolowo University, Ile-Ife, Nigeria; 3 Institute for Integrated Natural Sciences, University of Koblenz-Landau, 56070 Koblenz, Germany

**Keywords:** Chironomids, Damselfly, Mayfly, Gulf of Guinea, Rivers, Synthetic polymer

## Abstract

Plastic pollution has enormous impacts on freshwater and marine ecosystem health, and it is one of the topmost environmental concerns of the current geological period (i.e. the Anthropocene). Thus, the goal of our study was to provide baseline information and bridge the information gap on the occurrence of microplastics (MPs) in African freshwater systems, using two tributaries of the Gulf of Guinea (Ogun and Osun Rivers) in Nigeria as a case study and three freshwater insect species of different feeding guilds as bioindicators. A total of 29 individuals of the insect species were chemically digested and subsequently analysed for MP presence under a digital microscope and a micro-Fourier-transform infrared (μFTIR) spectroscope. Collector-gatherers (*Chironomus* sp. and *Siphlonurus* sp.) recorded the highest MP load per gram wet weight, while the predatory *Lestes viridis* recorded the lowest. The highest diversity of polymers was recorded in *Chironomus* sp. of Ogun River, i.e. styrene ethylene butylene styrene, acrylonitrile butadiene styrene (ABS), chlorinated polyethylene, polypropylene (PP), and polyester, while two polymers each were recorded in *Siphlonurus* sp. (i.e. polyester and ABS) and *L. viridis* (i.e. polyester and PP) of Osun River. We conclude that collector-gatherers like *Chironomus* sp. and *Siphlonurus* sp. could be best employed as MP bioindicators in freshwater systems. However, their suitability as MP bioindicators should be further investigated in different freshwater ecosystems worldwide.

**Figure:**
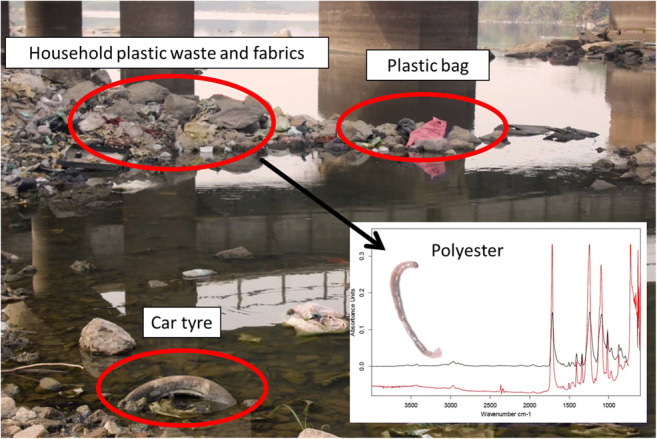
Graphical abstract

Graphical abstract

## Introduction

Reports on the ubiquity of microplastics (MPs) in the biosphere have been well documented, from alpine lakes to deep-sea sediments (e.g. Van Cauwenberghe et al. 2013; Free et al. 2014; Woodall et al. 2014) and from temperate to tropical environments (e.g. Mani et al. 2015; Horton et al. 2018; Nel et al. 2018; Akindele et al. 2019). Aside from primary sources (e.g. abrasives in skin cleansers, toothpaste, shaving cream and related products), another major pathway of MPs into aquatic systems of developing nations is through mechanical disintegration of large plastic debris (UNEP 2016). The introduction of such large plastic debris into aquatic systems of many developing nations is usually due to poor waste management practice (Akindele et al. 2019). Such plastics and MPs pose a great danger to wildlife and human populations when introduced into aquatic environments (Galloway 2015; Li et al. 2015; Carbery et al. 2018). Accumulation of MPs by aquatic animals, for instance, could endanger their populations through decreased food consumption, weight loss, decreased growth rate and energy depletion (GESAMP 2015; Rochman et al. 2015; Lusher et al. 2017).

There is evidence that MPs can be transferred from one trophic/biological level to higher levels up to human populations (Farrell and Nelson 2013; Hurley et al. 2017). Such particles have the tendency of being retained in the body of predators for a longer period, thus increasing their susceptibility to MP-related hazards (Lambert and Wagner 2018). Benthic macroinvertebrates have been copiously used in biomonitoring owing to the fact that most members are sessile and their lifespan is long enough for assessment of site-specific ecological conditions (Rosenberg and Resh 1993; Voshell 2002). Recently, there have been studies providing evidence of MP ingestion by freshwater macroinvertebrates. Among such reports are Hurley et al. (2017) on oligochaetes, Nel et al. (2018) on Diptera/chironomids, Akindele et al. (2019) on gastropods and Windsor et al. (2019) on Ephemeroptera and Trichoptera. Given the fact that freshwater macroinvertebrates have a wide range of feeding guilds (e.g. grazers, shredders, collectors-filterers, collector-gatherers and predators) and ecological niches (Voshell 2002), they could be suitable indicators for assessing MP pollution, both in the water column and in the benthic zone of lotic freshwater systems. High rate of MP ingestion by macroinvertebrates in some UK rivers was recently reported by Windsor et al. (2019), with approximately 50% of all sampled insects ingesting MPs, and they were recorded in three insect families (i.e. Heptageniidae, Baetidae and Hydropsychidae). Until now, there are two reports of MPs in African freshwater invertebrates (Nel et al. 2018 in South Africa; Akindele et al. 2019 in Nigeria), though only the latter reported MP polymers in their study. Microplastic ingestion by deposit feeders such as ephemeropterans and dipterans has recently been reported by some authors (e.g. Nel et al. 2018; Windsor et al. 2019), and invertebrates such as *Chironomus* sp. have been strongly recommended as MP bioindicators in freshwater systems (Nel et al. 2018; Scherer et al. 2018). In Nigeria, polyethylene bags have been reported as one of the causes of plastic pollution in aquatic systems, and their ingestion by a freshwater gastropod was most recently reported by Akindele et al. (2019) in Osun River. In view of the foregoing, this study aimed at giving a further insight into the presence and chemical nature (polymers) of MPs in two of the headwaters of the Gulf of Guinea in Nigeria (i.e. Ogun and Osun Rivers), using the representatives of the following aquatic insect orders as bioindicators: Odonata (*Lestes viridis*), Ephemeroptera (*Siphlonurus* sp.) and Diptera (*Chironomus* sp.). The three insect species are part of the benthic community in an aquatic environment, but they belong to two functional feeding guilds. *Chironomus* sp. and *Siphlonurus* sp. are collector-gatherers or deposit feeders, while *L. viridis* is a predator (Voshell 2002). Thus, the study also assessed MP presence and polymers based on two feeding guilds of aquatic invertebrates in the rivers. With respect to these insects, the study also seeks to find out which of the macroinvertebrate taxa or feeding guilds could be best employed as MP bioindicators in (African) freshwater systems.

## Materials and methods

### Study area

**Fig. 1:**
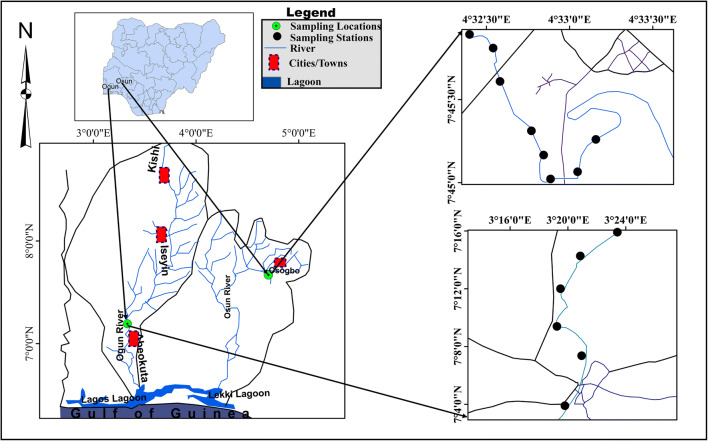
Map of the study area showing the sampling stations (inset a: map of Nigeria showing the locations of Osun and Ogun Rivers; inset b: map of Ogun and Osun River Basins showing their connections with the Gulf of Guinea

**Fig. 2:**
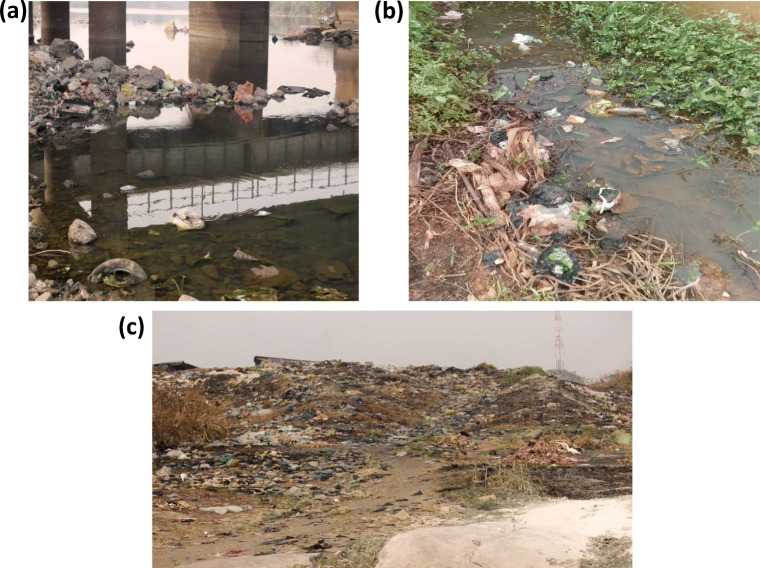
Evidences of macroplastic debris in the study area **a** littoral side of Ogun River **b**, littoral side of Osun River and **c** bank of Ogun River

The study was conducted in Ogun and Osun Rivers, the two major rivers in the southwestern part of Nigeria. The two rivers are both tributaries of the Gulf of Guinea within the Nigerian territory. Ogun River takes its source from Oyo State and flows through Ogun State into the Lagos Lagoon which borders with the Gulf of Guinea. On the other hand, Osun River takes its source from Ekiti State, flows through Osun, Oyo, and Ogun States, and finally flows into the Lekki Lagoon at Lagos State (Fig. 1). Although the Lekki Lagoon is connected to the Lagos Lagoon, it is not under tidal influence, unlike the Lagos Lagoon which is directly linked with the ocean. In summary, Ogun and Osun Rivers form a confluence at the Lagos Lagoon which in turn connects with the Gulf of Guinea. The two rivers are being jointly managed by the Ogun-Osun River Basin Development Authority (RBDA), which is one of the 12 RBDAs in Nigeria. In the study area of both rivers, there were evidences of large plastic debris in the rivers as well as on their banks, e.g. polyethylene bags, car tyres and fabrics (Fig. 2).

### Collection of samples

In October 2018 (peak of rainy season), aquatic insect samples were collected from the largest and capital cities of both states, i.e. at Abeokuta for Ogun River and Osogbo for Osun River (Fig. 1). Sampling effort was deployed over a stretch of approximately 3 km from the littoral/sub-littoral sections of each river using a pond net, from eight sampling points at Osun River and six stations at the Ogun River. Individuals of *Chironomus* sp. were collected from Ogun River, while *L. viridis* and *Siphlonurus* sp. were collected from Osun River, respectively. All samples were preserved in the field using 70% ethanol.

### Laboratory analysis

Based on the method described by Li et al. (2015) and Naji et al. (2018), five individuals of each species were pooled together to constitute a sample. For each species, two samples (*N* = 2) were prepared for analysis, thus consisting of ten individuals (*n* = 10) per species. The only exemption to this was in the case of *Siphlonurus* sp. where there were two samples of five and four individuals each. The wet weight of each insect was measured using an OHAUS weighing balance (Explorer EX 224). Prior to chemical digestion, insect samples were first rinsed with ultrapure distilled water to get rid of any potential laboratory contaminant, after which samples were immediately transferred into individual glass beakers under a fume cupboard. Twenty (20) ml each of KOH (10 M) and H_2_O_2_ (34.5–36.5% v/v) were added to each sample, and the beaker was covered with parafilm. The samples were thereafter shaken on a laboratory shaker (Edmund Buhler 7400 Tübingen) for 120 h. Each sample was neutralized with 7.78 ml formic acid and later filtered using an Anodisc™ 47 (also known as aluminium oxide) Whatman filter paper with 0.2-μm pore size. Each filter paper was oven-dried at 50 °C for 48 h, and it was thereafter viewed under the digital microscope system (VHX-2000 series, Osaka, Japan) for MP occurrence and abundance.

### Quality assurance/quality control measures

**Fig. 3:**
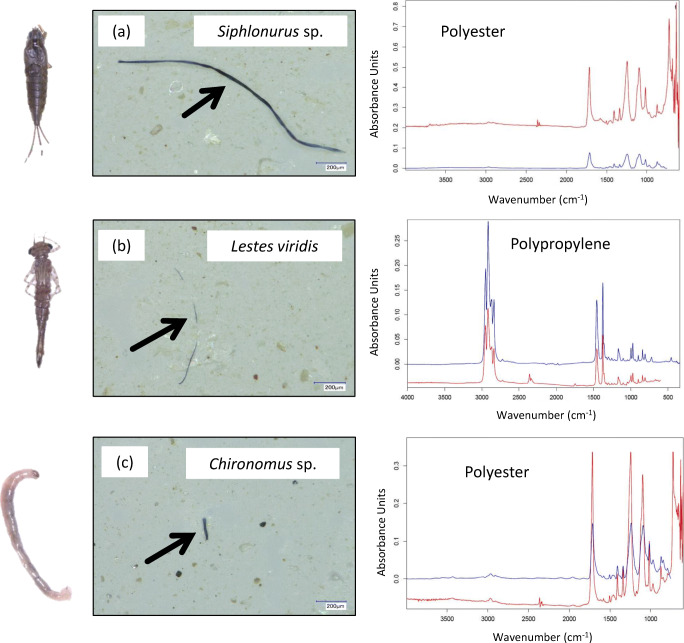
Representative microplastics in the freshwater insects of Osun and Ogun Rivers, Nigeria. **a** *Siphlonurus* sp., **b** *Lestes viridis* and **c** *Chironomus* sp. (The red spectrum is that of the μFTIR measurement, while the blue spectrum is the reference spectrum from the Bruker database)

At every stage of the analysis, a blank sample was also prepared by employing the same procedure used for the samples. In the same vein, MPs that co-occurred in any sample and the blank were considered to have been exogenously introduced, and such were taken out of the records. Confirmation of each suspected MP and MP polymer determination were done under the micro-Fourier-transform infrared (μFTIR) spectroscope (Hyperion 2000, Bruker, Ettlingen, Germany, see also Fig. 3). The measurements were performed in attenuated total reflectance (μATR) mode with 32 co-added scans and a spectral resolution of 4 cm^−1^. The software used for polymer identification was OPUS 7.5. Only particles with a hit quality of over 700 were considered as MPs (Ehlers et al. 2019). Soft nitril hand gloves and 100% cotton laboratory coats were worn during laboratory analysis. All apparatus used were also cleaned and rinsed with ultrapure distilled water at every stage of the analysis.

### Statistical analysis

As all species had different body sizes, we standardized their MP loads as the number of MPs per gramme wet weight for all three species. Then, we checked the data for normality using the Kolmogorov-Smirnov test and checked for homogeneity of variances using Cochran’s C test. As the assumptions for an ANOVA were met, we then performed a one-factorial ANOVA. All statistical tests were performed in Statistica 13 (Tulsa, OK, USA).

## Results

Comparatively, *Chironomus* sp. was the smallest insect in this study based on wet body weight which ranged from 5.6 to 17.5 mg. The wet body weight of *L. viridis* ranged from 17.7 to 33.8 mg. *Siphlonurus* sp. was the biggest and its weight ranged from 15.1 to 82.2 mg. Two types of microplastics were recorded in this study, namely, fibres and fragments. The two MP types were recorded in *Chironomus* and *Siphlonurus* sp., while only one (i.e. fibre) was recorded in *L. viridis* (Table 1). Fibre was recorded in all the species, and it dominated MP composition in each case. The least dominance (55.2%) of fibre was recorded in *Chironomus* sp. and the highest (100%) in *L. viridis*. Fragment, which occurred only in two species, recorded the lowest (17.4%) MP dominance in *Siphlonurus* sp., while the highest (44.8%) was in *Chironomus* sp.. The following trend was observed among the species with respect to MP load per wet weight: *Chironomus* sp. > *Siphlonurus* sp. > *L. viridis*. The ANOVA indicated a significant difference between the species regarding MP load per gram wet weight, i.e. F (2,3) = 22.14, *p* = 0.016. Tukey’s HSD showed that the MP load per gramme wet weight in *Chironomus* sp. was significantly different from the load in *Siphlonurus* sp. (*p* = 0.024) and *L. viridis (p* = 0.019), respectively. There was no difference in MP load per gramme wet weight between *L. viridis* and *Siphlonurus* sp. (*p* = 0.894). Images of some recorded MPs and their corresponding polymer spectra are shown in Fig. 3. Micro-FTIR analysis revealed the following MP polymers in Osun River: polyester (in both *L. viridis* and *Siphlonurus* sp.), polypropylene (in *L. viridis* only) and acrylonitrile butadiene styrene (ABS) (in *Siphlonurus* sp. only). In *Chironomus* sp. of Ogun River, the following MP polymers were recorded: styrene ethylene butylene styrene (SEBS), ABS, chlorinated polyethylene, polypropylene and polyester MPs.

**Table 1:** Microplastic loads (per gram wet weight) in the three insect species of Ogun and Osun Rivers, Nigeria

Species	N	*Number of pooled individuals	MP load per gram wet weight (mean ± SEM)		
			Fibre	Fragment	Total
*Lestes viridis*	2	*10 (5 + 5)	43.29 ± 43.29	0	43.29 ± 43.29
*Siphlonurus* sp.	2	*9 (5 + 4)	51.81 ± 1.13	10.54 ± 4.66	62.36 ± 3.53
*Chironomus* sp.	2	**10 (5 + 5)	179.43 ± 85.82	112.33 ± 59.28	291.76 ± 26.55

*Number of pooled individuals from the eight stations in Osun River; **Number of pooled individuals from the six stations in Ogun River

## Discussion

In this study, MPs were recorded in different orders of insects as well as different feeding guilds, thus suggesting that aquatic animals of different taxonomic categories or ecological niches may be predisposed to MP pollutants. *Chironomus* sp. had a significantly higher MP body burden per gramme wet weight than the other two species. *Chironomus* sp. is a collector-gatherer with the capacity to feed on deposited organic materials on stream or riverbeds (Voshell 2002; Nel et al. 2018). Unlike the water column, this section of an aquatic system has a higher retention capacity and is regarded as the most important sink of pollutants in freshwater environments (Morin et al. 2007). Primary or secondary MPs could end up in any of the following ways in river systems: (1) they could drift with the water mass into adjacent oceans or lakes; (2) like other suspended solids, they could settle on the riverbed when flow velocity is too low to keep them in suspension (Voshell 2002); and (3) they could outrightly be ingested by aquatic animals as previously reported by several authors (e.g. Rosenkranz et al. 2009; Akindele et al. 2019; Windsor et al. 2019). The suitability of deposit feeders, particularly *Chironomus* sp., as MP bioindicator has also been reported by Nel et al. (2018) in a South African river system. Deposit feeders may therefore be suitable as MP bioindicators in lotic freshwater systems since they are not only site-specific, but they can also indicate impacts over a period of time. In previous lab experiments, it has been shown that MP ingestion can affect the life history (Silva et al. 2019) and survival, growth and emergence (Ziajahromi et al. 2018) in chironomid larvae. Hence, those insects may also be negatively impacted by MP in the field. Other evidences of low physiological fitness in aquatic invertebrates on account of MPs include reduced filtering or feeding capacity in blue mussel (*Mytilus edulis*) and reduced reproductive output in a species of copepod (*Calanus helgolandicus*) (Wegner et al. 2012; Cole et al. 2013). Ingested MPs in aquatic animals could also lead to transfer of hydrophobic and persistent organic pollutants (e.g. dichlorodiphenyltrichloroethane, polychlorinated biphenyls and dioxins) to higher trophic levels in the food chain, since MPs serve as vectors for such transfer (Teuten et al. 2009). Thus, MPs could pose a threat to the sustenance of aquatic biodiversity considering their physiological and ecotoxicological implications when ingested by animals.

Like in many MP studies, fibres dominated MP composition in both rivers and the three insects. Among other causal factors, Browne et al. (2011) reported that a key important source of microplastic fibres appears to be through sewage contaminated by fibres from washing clothes. They also reported that a garment can produce > 1900 fibres per wash or > 100 fibres per litre of effluent. Incidentally, polyester occurred in both rivers, and this has been linked to sources such as clothing, automotive tyre cords and beverage containers (Harper 1996; Gowariker et al. 2001). Introduction of polyester MP could be linked to direct washing of clothes inside streams and rivers which is a common practice by many locals in many parts of Nigeria and Africa, due to poor economies and lack of domestic water supply, especially in rural and semi-urban areas. In addition, evidence of worn-out tyres was also sighted inside Ogun River from which the *Chironomus* sp. was collected (Fig. 2a). Sources of other MP polymers recorded in this study can also be related to prevalent plastic sources in Nigeria. These include the following: polypropylene, e.g. fabrics, biscuit wrappers, rope, bottle caps and drinking straws; ABS, e.g. highway safety devices and toys; SEBS, e.g. toy products, shoe soles, road paving and roofing applications; and chlorinated polyethylene which is often used in conjunction with polyvinyl chloride and has a wide range of applications in construction (Harper 1996; Gowariker et al. 2001; GESAMP 2015). The heterogeneity of MP types recorded in these rivers as well as the insects could be a reflection of various applications of plastics in the respective river basins. Going by the level of plastic deposits on the riverbanks as well as in the rivers, it is also most likely that these MPs are mostly derived secondarily through fragmentation of larger plastic debris and through factors such as wind, ultraviolet radiation and animal digestion (UNEP 2016).

In conclusion, this is the first study to show that MPs can be ingested by larval odonatans, insects that are threatened in many countries worldwide. Collector-gatherers seemed to record more diverse polymers than predatory insects in this study. However, future studies should seek to test further if collector-gatherers (e.g. *Chironomus* sp. and *Siphlonurus* sp.) could be best employed as MP bioindicators among other macroinvertebrate taxa, by using a larger sample size. It would also be important to consider MP analysis in both water columns as well as in riverbeds, from which MPs are ingested by the animals.
